# The international and domestic research trends of targeted therapy in bladder cancer: a bibliometric analysis of the past three decades

**DOI:** 10.3389/fonc.2026.1850175

**Published:** 2026-05-25

**Authors:** Yijin Pan, Yu Sun, Binbin Jiao, Yinqiang Zhang, Guan Zhang

**Affiliations:** 1 Department of Nuclear Medicine, Peking Union Medical College Hospital, Peking Union Medical College, Chinese Academy of Medical Sciences, Beijing, China; 2 Department of Colorectal Surgery, Peking Union Medical College Hospital, Peking Union Medical College, Chinese Academy of Medical Sciences, Beijing, China; 3 Department of Urology, Beijing Chao-Yang Hospital, Capital Medical University, Beijing, China; 4 Department of Urology, China-Japan Friendship Hospital, Beijing, China

**Keywords:** bibliometrics, bladder cancer, CiteSpace, data visualization, targeted therapy

## Abstract

**Introduction:**

With the rapid development of molecular biotechnology and in-depth exploration of bladder cancer (BC) oncogenic mechanisms, targeted therapy has emerged as a pivotal treatment modality for BC patients. This study employed bibliometric methods to analyze the research status and development trends of targeted therapy in BC both domestically and internationally, aiming to identify research hotspots and provide a reference for research layout in this area.

**Methods:**

A systematic search was conducted across renowned international and domestic databases, including Web of Science Core Collection (WoSCC), PubMed, Wanfang Database, and the Chinese National Knowledge Infrastructure (CNKI). The search period was limited to 1994–2024, with search terms including “bladder cancer”, “targeted therapy”, and their related derivatives. After screening, 912 English publications and 328 Chinese publications were included. WPS Excel (v12.1.0) was used to visualize trends in annual publication volume, and CiteSpace (v6.3.R1) was employed for a visual analysis of countries/regions, institutions, authors, journals, cited articles, and keywords related to the included publications.

**Results:**

In the field of targeted therapy in BC, the annual publication volume has generally shown an upward trend over the past three decades for both English and Chinese publications, with a faster growth rate observed in English publications. For English publications, the most productive countries/regions, institutions, authors, and journals were the USA, University of Texas System, Dinney CPN, and Urologic Oncology—Seminars and Original Investigations, respectively. The most cited article was “Comprehensive Molecular Characterization of Urothelial Bladder Carcinoma” published in *Nature*. A cluster analysis of keywords revealed research hotspots such as “phase-ii trial”, “cell line”, and “EGF receptor”. For Chinese publications, the most productive institutions, authors, and journals were the Second Hospital of Jilin University, Wang JS, and Chinese Journal of Urology, respectively. The most cited article was “Advances in the Treatment of High-Risk Non-Muscle-Invasive Bladder Cancer” published in Shandong Medical Journal. The cluster analysis of keywords identified research hotspots including “migration”, “cell proliferation”, and “drug resistance”.

**Discussion:**

Currently, Europe and America dominate the field of targeted therapy in BC. China can leverage its advantage in terms of patient population size to strengthen domestic and international collaborations, thereby enhancing its research influence in this field. Global research hotspots reveal the translational process of targeted therapy in BC from bench to bedside. Future research in this field will focus on drug management of targeted therapy for metastatic BC and overcoming drug resistance to targeted therapy.

## 1 Introduction

Bladder cancer (BC) is one of the most common malignancies of the urinary system with significant morbidity and mortality rates (1). BC is categorized into two main types depending on the depth of tumor infiltration: non-muscle-invasive bladder cancer (NMIBC) and muscle-invasive bladder cancer (MIBC) (2). MIBC accounts for approximately 25% of all BC cases and is associated with a poor prognosis. The remaining 75% are classified as NMIBC, which is characterized by a significant recurrence rate (3). Despite numerous advancements in the diagnosis and treatment of BC in recent years, the survival rate for BC patients has shown only modest improvements (4). Furthermore, the economic burden of managing this disease remains considerable, driven by the high frequency of recurrence and the need for lifelong surveillance (5). Therefore, a comprehensive analysis of the pathological mechanisms and exploring novel therapeutic approaches for BC are essential for improving the prognosis of BC patients.

Targeted therapy represents an emerging approach to cancer treatment that aims to inhibit tumor growth by specifically targeting key molecular sites within cancer cells while minimizing damage to normal tissues (6). With deeper understanding of the biological behavior of BC, researchers have identified multiple potential therapeutic targets. Notably, the pan-FGFR tyrosine kinase inhibitor erdafitinib and the anti-Nectin-4 antibody–drug conjugate enfortumab vedotin have recently received FDA approval in the United States (7, 8). Besides that, recent progress in elucidating the molecular characteristics of BC has provided a theoretical foundation for the development of effective targeted drugs for BC and have spurred a series of related studies.

However, to fully comprehend the research status and development trends in the field of targeted therapy for BC, it is essential to focus on the latest scientific breakthroughs from a macro perspective. Bibliometric analysis, as a quantitative research method, can reveal research trends and contributions of countries, institutions, and individuals in a specific field by statistically analyzing publication numbers, citation frequencies, and collaboration networks under a particular theme (9). This method helps identify research hotspots, frontier directions, and developmental dynamics within the field, thereby guiding future research.

Therefore, this study intends to employ bibliometric methods to comprehensively analyze English and Chinese publications about targeted therapy for BC from 1994 to 2024. The objective of this study is to gain a deeper understanding of the research background, developmental trajectory, and potential research trends in this field, providing reference for further exploration of precision treatment strategies for BC.

## 2 Materials and methods

### 2.1 Data sources

Web of Science and PubMed are authoritative citation index databases offering access to the latest medical literature on a worldwide scale. For this study, English publications were extracted from the Web of Science Core Collection (WoSCC) and PubMed using “advanced search”, while Chinese publications were obtained from Wanfang and the Chinese National Knowledge Infrastructure (CNKI), two commonly used databases for checking domestic medical literature, through the function of “professional search”.

### 2.2 Search strategies

The search timeframes for all four databases were limited to the period from May 1, 1994 to May 1, 2024. Specific retrieval strategies were implemented as follows:

PubMed: ((bladder[Title]) AND (Neoplasia*[Title] OR malignan*[Title] OR cancer*[Title] OR carcinoma*[Title] OR tumor*[Title] OR tumour*[Title])) AND (“targeted therap*”[Title/Abstract] OR “kinase inhibitor*”[Title/Abstract] OR “molecular target*”[Title/Abstract]), text availability=“full text”, article language= “English”

WoSCC: TI=(bladder) AND TI=(Neoplasia* OR malignan* OR cancer* OR carcinoma* OR tumor* OR tumour*) AND TS=(“targeted therap*” OR “kinase inhibitor*” OR “molecular target*”), literature type=“article or review article”, article language= “English”

Wanfang: Title: (bladder cancer OR bladder tumor) AND Subject: (targeted therapy OR molecular target OR kinase inhibitor), literature type = “journal article”, text availability=“full text”;

CNKI: TI=(bladder cancer OR bladder tumor) AND TKA=(targeted therapy OR molecular target OR kinase inhibitor), literature type=“journal article”

### 2.3 Screening process

**Figure 1:**
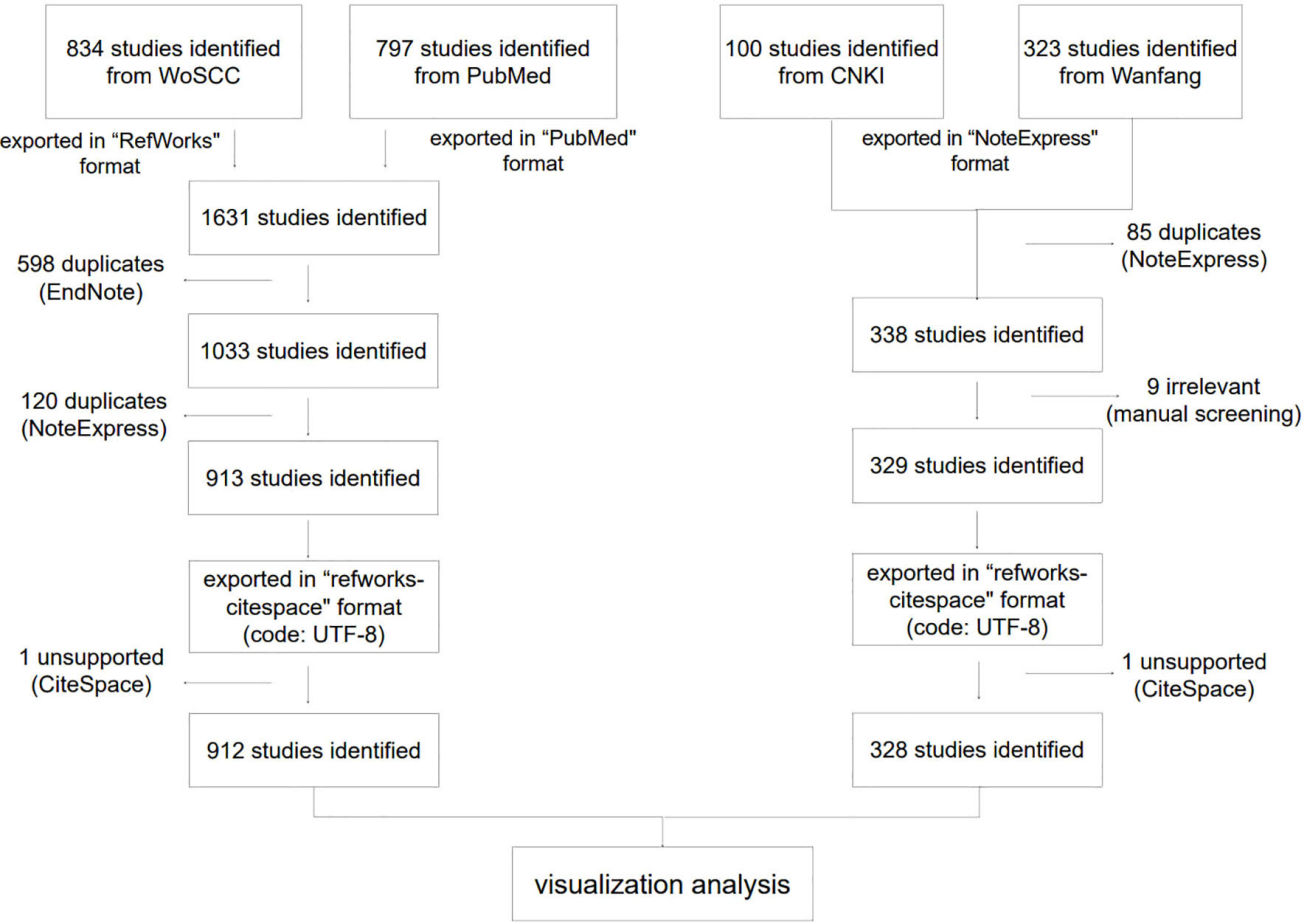
Flowchart of the study.

The Chinese publications were sourced from Wanfang and CNKI and then imported into NoteExpress (v4.0.0) in “RefWorks” format for initial deduplication. Refined publications were exported in “RefWorks-CiteSpace” format (UTF-8 encoded, filenames “download_#.txt”) and further deduplicated using CiteSpace (v6.3.1R). The English publications from WoSCC and PubMed underwent initial deduplication in EndNote (v20.6). After importing into NoteExpress in “EndNote export” format, similar procedures were followed as for the Chinese publications. Two independent researchers screened titles and abstracts and excluded irrelevant papers through cross-checking. Ultimately, 912 English and 328 Chinese publications were included for analysis. The flowchart of this study is shown in **Figure 1**.

### 2.4 Visualization tools

For visualization analysis, WPS Excel (v12.1.0) was used to create a chart depicting annual publication trends and tables ranking country/region, institution, author, journal, and references. Other visualizations, including collaboration networks of institutions or authors, citation bursts of authors or keywords, cluster maps, and timeline charts of keywords, were generated by using CiteSpace (v6.3.R1) (10). The time slice and the “top *N*% per slice” were set to 1 year and 50%, indicating that nodes with the top 50% of highest frequencies were retained for subsequent analysis within each year. Notably, latent semantic indexing (LSI) and log-likelihood ratio (LLR) algorithms were employed for clustering English and Chinese keywords, respectively. The remaining values were set to their defaults.

### 2.5 Important concepts

The minimal number of publications for core authors is determined by using Price’s Law, which is formulated as $N=0.749\times \sqrt{\max}$, where “max” denotes the number of publications by the most productive author. In terms of journal-related concepts, the impact factor (IF) and journal citation reports (JCR) quartile are extracted from WoSCC to depict journals of English publications, with journals in the first quartile (Q1) generally regarded as excellent journals. Journals included in the Chinese Science and Technology Paper Citation Database (CSTPCD) are regarded as leading journals of Chinese publications. Modularity (*Q* value) and weighted mean silhouette (*S* value) are used to assess the performance of clustering. The *Q* value ranges from 0 to 1, with a value exceeding 0.3 signifying a significant clustering structure. The *S* value ranges from -1 to 1, with a value exceeding 0.5 denoting that the results of the clusters are reasonable, while the results are highly reliable when the value exceeds 0.7. Furthermore, betweenness centrality was employed to measure the ability of one node to connect with other nodes, with a centrality value of ≥0.1 indicating an important status of this node in the collaboration network (11).

## 3 Results

In this study, a total of 912 English publications from 768 authors affiliated with 587 institutions were included, while Chinese publications were relatively fewer, with a total of 328 publications from 509 authors affiliated with 297 institutions being retrieved.

### 3.1 Trends in annual publication volume

**Figure 2:**
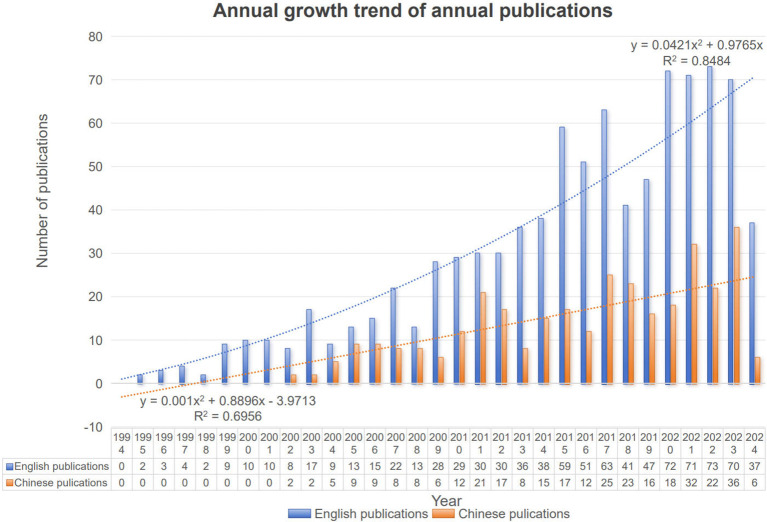
Annual publication trends from 1994 to 2024. Trends in the annual publications on targeted therapy in bladder cancer between 1994 and 2024 (blue: English publications, orange: Chinese publications).

**Figure 2** provides a comprehensive view of the trends in annual publication volumes for English and Chinese publications from 1994 to 2024. For English publications, the number of annual publications grew slowly during the initial period, as evidenced by the first recorded two in 1995 and remaining below 10 until 2000. Subsequently, the number increased more rapidly, and a surge was seen between 2015 and 2017. Although publication activity between 2018 and 2019 was lower compared to the preceding 3 years, a new surge in research activity has been observed since 2020.

The total number of Chinese publications was lower compared to English publications, which may be partly attributed to a later start and a comparatively slower growth rate. The number of annual publications was first recorded in 2002 and gradually increased afterward. The peak was observed in 2023 with 36 publications—approximately half of the number observed in English publications (73). Notably, the difference in trends of annual publication volume observed between the two languages may be partly due to some studies originally conducted in Chinese being published in English.

### 3.2 Country/region and institution analyses

Research output is an important metric which can reflect the research activity and contributions of a country, an institution, or an author in one specific field. Detailed information on the top 10 most productive countries in English publications is shown in **Table 1**. The collective output of these countries accounted for 63.13% (1,541/2,441) of the total English publications, with two countries having outputs exceeding 100—USA (268) and China (149). Although Japan ranked third, its output (39) exhibited a significant gap compared to the top two. Notably, USA (0.68) and China (0.12) not only dominated in the output but also emerged as significant collaboration centers among countries publishing English publications in this field, as evidenced by their centrality values surpassing 0.1.

**Table 1:** Top 10 most productive countries/regions and institutions in English and Chinese publications within this field.

	English (WoSCC and PubMed)						Chinese (CNKI and Wanfang)		
Rank	Country/region	Frequency	Centrality	Institution	Frequency	Centrality	Institution	Frequency	Centrality
1	USA	268	0.68	University of Texas System	68	0.1	Second Hospital of Jilin University	13	0.00
2	People’s Republic of China	149	0.12	University of California System	41	0.02	Second Affiliated Hospital of Kunming Medical University	11	0.00
3	Japan	39	0.06	Indiana University System	22	0.02	First Affiliated Hospital of Shanxi Medical University	7	0.00
4	Germany	21	0	Assistance Publique Hopitaux Paris (APHP)	17	0.1	First Hospital of Peking University	6	0.00
5	England	21	0	Harvard University	15	0.01	First Affiliated Hospital of Chongqing Medical University	6	0.00
6	Canada	20	0	Sun Yat-sen University	14	0	Tongji Hospital, Tongji Medical College of Huazhong University of Science and Technology	6	0.00
7	Greece	9	0.01	Centre National de la Recherche Scientifique (CNRS)	13	0.01	First Affiliated Hospital of Soochow University	5	0.00
8	India	6	0	Cornell University	12	0.01	Shanxi Medical University	5	0.00
9	Poland	5	0	Baylor College of Medicine	11	0.03	Union Hospital, Tongji Medical College of Huazhong University of Science and Technology	5	0.00
10	Switzerland	5	0	Memorial Sloan Kettering Cancer Center	10	0.01	Fifth Affiliated Hospital of Sun Yat-sen University	4	0.00

Regarding the top 10 most productive institutions contributing to English publications, the University of Texas System (68), University of California System (41), and Indiana University System (22) occupied the top three positions. The majority of these 10 institutions are from European and American areas, with the domestic institution Sun Yat-sen University ranking sixth (14). The University of Texas System (0.1) and Assistance Publique Hôpitaux de Paris (0.1) are notable for their centrality values exceeding 0.1, highlighting their important role in institutional cooperation and communication for English publications in this field. For Chinese publications, the top 10 most productive institutions exhibit a wide geographical distribution across China, with the Second Hospital of Jilin University (13), the Second Affiliated Hospital of Kunming Medical University (11), and The First Affiliated Hospital of Shanxi Medical University (7) being the top three. However, no institution stands out as a collaboration center within Chinese publications, given that the centrality values of institutions involved are all below 0.1 (**Table 1**).

### 3.3 Author analysis

**Figure 3:**
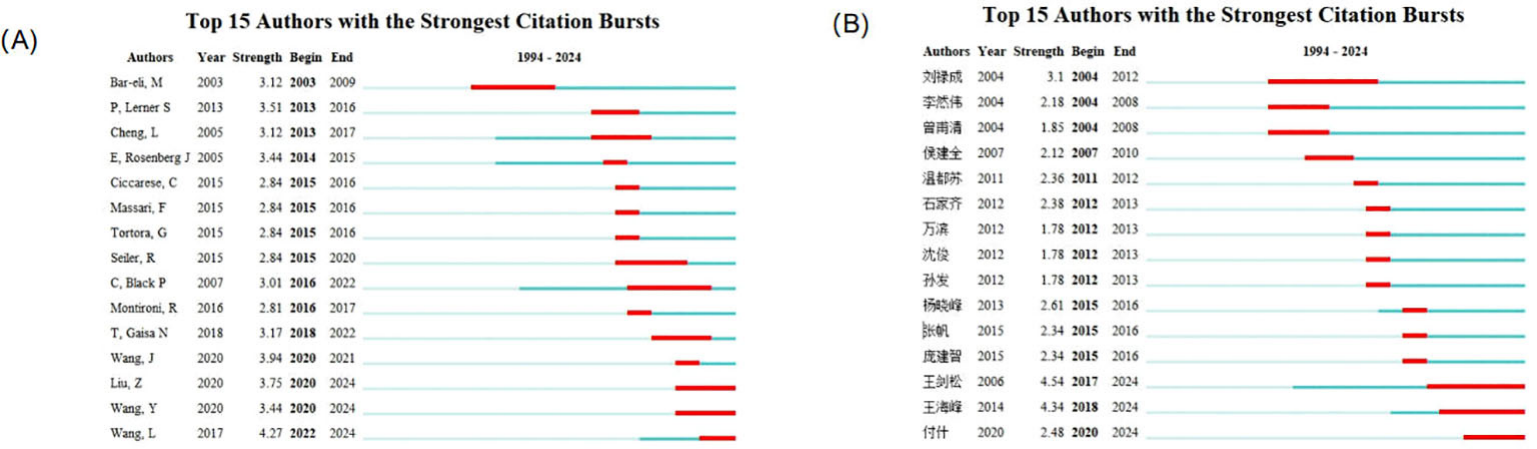
Author analysis within the field. **(A)** Top 15 burst authors in English publications. **(B)** Top 15 burst authors in Chinese publications. Blue lines indicate time intervals, and red lines indicate active periods.

**Table 2** presents the top 10 most prolific authors in English and Chinese publications on targeted therapy in BC. For authors who published English publications, the majority were affiliated with medical centers and universities in North America and Europe. The top 10 most productive authors demonstrated great productivity and impact, with H-index scores ranging from 27 to 85. Among these 10 authors, Dinney CPN led with 20 publications, followed by Cheng L with 16, and McConkey DJ, Black P, and Knowles MA, each with 12. The threshold for core authors was set at four publications by Price’s Law, and 8.20% (63/768) of the authors were eventually identified, with approximately one-quarter of them being Chinese researchers. Author burst analysis (**Figure 3A**) revealed that Bar-eli M and Black P had the most sustained activity at 6 years, suggesting their consistent and stable research output. The active periods of Chinese researchers Liu Z, Wang Y, and Wang L extended to 2024, suggesting that their work has gained extensive attention in recent years. Although Dinney C held the highest centrality value at 0.04, this figure was below 0.1 and suggested that a central figure in collaboration had yet to emerge.

**Table 2:** Top 10 most productive authors in English and Chinese publications within this field.

English (WoSCC and PubMed)				Chinese (CNKI and Wanfang)		
Author	Frequency	Institution	H-index	Author	Frequency	Institution
Dinney CPN	20	UTMD Anderson Cancer Center	85	JS Wang	18	Second Affiliated Hospital of Kunming Medical University
Cheng L	16	University of British Columbia	71	HF Wang	15	Second Affiliated Hospital of Kunming Medical University
McConkey DJ	12	Johns Hopkins University	92	LC Liu	12	Second Hospital of Jilin University
Black P	12	University of British Columbia	59	XF Yang	8	First Affiliated Hospital of Shanxi Medical University
Knowles MA	12	University of Leeds	71	RW Li	7	Second Hospital of Jilin University
Theodorescu D	11	Cedars Sinai Medical Center	73	FQ Zeng	5	Union Hospital, Tongji Medical College of Huazhong University of Science and Technology
Pan CX	10	Harvard Medical School	31	M Zhang	5	Second Hospital of Jilin University
Rosenberg JE	10	Memorial Sloan Kettering Cancer Center	62	MX Ding	5	Second Affiliated Hospital of Kunming Medical University
Radvanyi F	10	Universite Paris Cite	57	S Fu	5	Second Affiliated Hospital of Kunming Medical University
Gaisa N	10	RWTH Aachen University Hospital	27	JQ Shi	4	Affiliated Hospital of Guizhou Medical Universit

Similar to English publications, the top 10 most productive authors in Chinese publications were predominantly from affiliated hospitals of Chinese universities, with The Second Affiliated Hospital of Kunming Medical University and The Second Hospital of Jilin University each contributing three out of the top 10 authors. Among these 10 authors, Wang JS led with 18 publications, followed by Wang HF (15) and Liu LC (12). The threshold for identifying core authors was set at three Chinese publications utilizing Price’s Law, and 7.27% (37/509) of the authors were screened ultimately. The analysis of author burst (**Figure 3B**) revealed that Liu LC maintained the longest period of sustained activity at 8 years. The authors whose active period extends to 2024 are Wang HF, Fu S, and Wang JS, suggesting a recent shift in focus toward their work. Notably, Wang JS exhibited the strongest burst strength at 4.54, which further highlighted the influence of his work in Chinese publications in this field. The centrality values of these 10 authors were all below 0.1, indicating a dispersed rather than centralized contribution among these authors.

### 3.4 Journal and cited article analyses

**Supplementary Table 1** delineates the top 10 most productive journals in English and Chinese publications, respectively. For English publications, the majority of these journals were categorized under oncology (5/10) and urology and nephrology (3/10) sections. The remaining two journals consist of one in the biochemistry and molecular biology section, while the classification of the other could not be confirmed due to its removal from the SCI database. Eight out of 10 of these journals were in the Q1 or Q2 JCR divisions, with IF ranging from 2.7 to 11.5, indicating that quality journals have a stronger appeal for publishing. Among these journals, the *Urologic Oncology-Seminars and Original Investigations* led with 32, followed by *Oncotarget* (19), *Cancers* (18), and *International Journal of Molecular Sciences* (18). The top 10 most-cited English publications were published in prestigious medical journals such as *Nature*, *The Lancet*, and *The Journal of the American Medical Association* (JAMA), underscoring their high academic quality and broad influence. The “Comprehensive Molecular Characterization of Urothelial Bladder Carcinoma”, which was published in *The Lancet* in 2014, received the highest number of citations (2,178). This study provided a comprehensive molecular landscape of BC by whole-genome and RNA sequencing, with the aim of identifying effective targeted agents for patients with BC (**Supplementary Table 2**).

For Chinese publications, the top 10 most productive journals have been influential within the Chinese academic community, as evidenced by their inclusion in the CSTPCD database. The overall output of these journals contributed 37.50% (123/509) of the total. The *Chinese Journal of Urology* topped the list with 22 articles, followed by the *Chinese Journal of Experimental Surgery* with 18. The *International Journal of Urology and Nephrology* and the *Journal of Modern Urology* tied for third place with 15 articles each. Among the top 10 most-cited Chinese publications, three were published in the *Journal of Medical Research & Combat Trauma Care*. This may indicate the great interest and contributions of this journal in this field. The article titled “Advances in the Treatment of High-Risk Non-Muscle-Invasive Bladder Cancer (NMIBC)”, published in *Shandong Medical Journal* in 2017, got the most number of citations (26). In this study, the research advances in various treatment modalities for high-risk NMIBC were reviewed, and targeted therapy was elaborated in the section on precision treatment (**Supplementary Table 2**).

### 3.5 Keyword analysis

Cluster analysis of keywords refers to categorizing keywords of similar topics into multiple clusters. Each cluster generates a specific label based on the common characteristics of the keywords within, which helps to analyze hotspots and research trends in this field. For English publications, 12 keyword clusters were ultimately formed utilizing the LSI algorithm. The meaningful clusters included “phase-ii trial” (#0), “cell line” (#4), “EGF receptor” (#6), “research & experimental” (#7), “aged” (#8), “cyclin D1/metabolism” (#9), “FGFR3 mutations” (#10), and “fibroblast growth factor” (#11) after excluding search terms like “bladder carcinoma” or “targeted therapy”. The total modularity *Q* value (0.5047) and the weighted mean *S* value (0.8023) of the clusters indicated a significant cluster structure and highly reliable cluster results (**Supplementary Figure 1A**). The timeline chart further elucidated a shift in research focus over the years. The initial period of this field was characterized by extensive exploration of disease mechanisms to identify effective targets for therapy, denoted by labels such as “FGFR3 mutations” and “fibroblast growth factor.” However, the emergence of labels related to clinical trials, such as “phase-II trials”, indicated a growing focus on the practical application of targeted therapy in clinical settings (**Supplementary Figure 1C**). Regarding the results of keywords burst analysis, “EGF receptor” has the most sustained active period at 12 years, followed closely by “gene amplification” and “colony-stimulating factor” at 9 years. These labels indicated that considerable efforts have been made over an extended period to develop therapies that effectively target these specific receptors and pathways. The remaining labels from keywords burst analysis included “dependent kinase inhibitor,” “tyrosine kinase inhibitor,” “transitional-cell carcinoma,” “growth-factor receptor,” “cisplatin-ineligible patients,” “biochemistry & molecular biology,” and “tumor microenvironment”. Notably, research focusing on the tumor microenvironment within the realms of biochemistry and molecular biology has garnered significant attention in recent years (**Supplementary Figure 1E**).

For keyword clusters of Chinese publications, the total modularity *Q* value (0.5475) and the weighted mean *S* value (0.9169) were relatively higher than those in English publications, indicating a more significant cluster structure and more reliable clusters results. A total of 10 keyword clusters were generated using the LLR algorithm, with clusters including “migration” (#3), “immunotherapy” (#5), “cell proliferation” (#6), “drug resistance” (#8), and “magnetic targeting” (#9) remaining after excluding search terms (**Supplementary Figure 1B**). Similar to those in English publications, these clusters covered both clinical and basic research aspects, with new concepts such as “drug resistance” and the combination of targeted therapy with “immunotherapy” being involved. Furthermore, the keywords burst analysis revealed that the active periods of “prognosis” and “cell proliferation” extend to 2024, underscoring the necessity for ongoing research into the prognosis of patients’ post-targeted therapy and the assessment of drug efficacy by inhibiting cell proliferation (**Supplementary Figure 1F**). The remaining labels from the keywords burst included “cell cycle,” “gene,” “apoptosis,” “nude mouse,” “pirarubicin,” “cell invasion,” “migration,” and “apoptosis”.

## 4 Discussion

BC ranks as the 10th most prevalent malignancy worldwide, with an estimated 500,000 new diagnoses and 200,000 deaths annually (12). BC is also one of the most economically challenging malignancies to manage throughout a patient’s lifetime, primarily due to the high recurrence and progression rate (13). The treatment of BC has seen significant advancements in recent years. While chemotherapy remains a cornerstone of treatment, its efficacy is far from satisfactory due to side effects and drug resistance. Targeted therapies, which utilize novel agents aimed at specific molecular pathways, present promising new opportunities to enhance outcomes (14). To gain further understanding of this field, we systematically examined both English and Chinese literature and revealed several key trends and areas of focus within the field. This study represented the first bibliometric analysis focused on targeted therapies for BC, providing a comprehensive overview of research trends and developments within this field. The findings highlighted the potential of targeted therapies in improving patient outcomes and identified the critical role of bibliometric analysis in mapping the trajectory of scientific progress.

As can be seen from the results, there is a continuous rise in the number of studies on BC-targeted therapy. From 2015 to 2017, the number of English publications on targeted therapies for BC saw a significant increase. Although there was a decline during 2018–2019, the publication count surged again in 2020 and has since remained at a high level. The number of related articles in Chinese has also been steadily increasing, reaching a peak in 2023. This indicates that this research area is receiving increasing attention from researchers around the world.

As illustrated in this study, the USA and China stand out with English publications, far exceeding those of other nations. Moreover, the high centrality values of the USA and China indicate their pivotal roles as collaboration hubs, suggesting their extensive engagement in international research networks. Influential research findings are predominantly concentrated among a select few institutions, and most of them are based in the USA and Europe. The centrality values of the University of Texas System and APHP further confirm their pivotal roles in institutional cooperation and communication. The majority of influential authors are also affiliated with leading medical centers and universities, indicating significant scholarly contributions. As for Chinese publications, the top 10 most productive institutions are geographically dispersed across China. No single institution emerges as a dominant collaboration center within Chinese publications. The top 10 most productive authors in Chinese publications are predominantly from affiliated hospitals of Chinese universities. However, the centrality values of the top 10 authors in both English and Chinese publications were all below 0.1, suggesting a dispersed rather than centralized contribution pattern. This result indicates that while the USA and China dominate both in terms of publication output and collaborative leadership, there remains significant potential for other countries and institutions to contribute and integrate into global research networks. Furthermore, fostering stronger cooperative ties within China could amplify its impact on the global stage, driving forward advancements in targeted therapies for BC.

The analysis of the most productive and the most cited journals in both English and Chinese publications on targeted therapies for bladder cancer reveals significant insights into academic impact and research quality. The majority of the English articles are published in the Q1 or Q2 JCR divisions, highlighting a preference for high-quality outlets. Leading Chinese journals contribute significantly to the national academic discourse and play a pivotal role in disseminating localized advancements and addressing region-specific challenges. Although the citation counts are modest compared to international counterparts, some impactful studies also demonstrate a growing interest within China.

The analysis of keywords provides valuable insights into the current research hotspots, highlighting the areas of greatest interest and activity within the field. The keywords identified in the English literature frequently focus on specific therapeutic targets such as FGFR, EGFR, and CDK. Alterations in the FGFR, especially FGFR3 mutations, are among the most common genetic changes in BC. These alterations are observed in approximately 60% of NMIBC and at least 15% of MIBC (15). Several FGFR inhibitors have been investigated or are currently under development, but the clinical effectiveness of these inhibitors is constrained due to primary and acquired drug resistance (16). Overexpression of EGFR can promote tumor progression by activating downstream oncogenic pathways, which is associated with poor prognosis in BC. Studies using cell and animal models have confirmed that EGFR inactivation not only inhibits the growth of BC but also increases the sensitivity to radiotherapy (17). Moreover, the combination of erdafitinib and gefitinib was effective in overcoming bypass resistance mediated by EGFR activation (18). Although several studies have indicated the potential of anti-EGFR therapy from a molecular mechanism perspective, many clinical trials utilizing EGFR inhibitors have not demonstrated additional benefits over standard chemotherapy in adjuvant or second-line treatments. A neoadjuvant study employing erlotinib prior to radical cystectomy has shown promising outcomes (19). Cyclin-dependent kinases (CDK) are proteins that integrate various signaling events triggering a cell to enter the mitotic cell cycle (20). A large proportion of BC shows molecular alterations in the cell cycle pathway, making the targeting of CDK 4 and 6 (CDK4/6) a promising therapeutic approach (21). A preclinical study showed that CDK4/6 inhibitor exerted antitumor effects *in vitro* and *in vivo*, suggesting the potential for the clinical management of BC patients (22). For Chinese publications, researchers pay a lot of attention to tumor biological behavior such as proliferation, invasion, and apoptosis. This focus reflects a strategic approach to tackling BC by targeting fundamental processes that drive tumor progression and metastasis. Furthermore, these keywords also reflect that research on targeted therapy for BC in Chinese literature mostly involves basic research on molecular targeting. From highly cited literature, these studies cover multiple directions such as suicide gene therapy, small interfering RNA (siRNA), and specific binding peptides—for example, as early as 2005, scholars constructed an adenovirus-mediated suicide gene system regulated by the human telomerase reverse transcriptase (hTERT) promoter and found that this system exerted targeted killing effects on human BC cells without significant damage to normal fibroblasts (23). In 2008, a study transfected small interfering RNA (siRNA) targeting survivin and found that survivin–siRNA could significantly downregulate survivin gene expression in BC cells, promote cell apoptosis, and inhibit tumor cell proliferation and the growth of xenografts *in vivo* (24). A 2015 study screened specific binding peptides for human bladder transitional cell carcinoma using *in vivo* phage display technology and found that the screened peptide NYZL1 could specifically bind to BC cells and tissues (25). More recent highly cited literature indicates that research on miRNA regulatory mechanisms has also been extensively conducted. In 2017, Lyu et al. (26) conducted miRNA omics analysis and cell function experiments and found that the inhibition of miR-130b-3p could upregulate PTEN expression and inactivate the PI3K-AKT and integrin β1/FAK signaling pathways, thereby inhibiting BC cell proliferation, migration, and invasion. Similarly, Zhang et al. (27) detected clinical samples and conducted cell experiments and found that miR-372-3p was lowly expressed in BC tissues and could inhibit BC cell proliferation, migration, and invasion by targeting ATAD2. These studies have explored potential molecular targets for BC diagnosis and treatment, providing a theoretical basis for the development of targeted therapeutic drugs for BC. Scholars have systematically summarized the treatment strategies for BC in two recently published reviews, including cutting-edge regimens such as targeted therapy, providing clear guidance for understanding the current status of BC treatment (28, 29).

BC-targeted therapy still faces multiple bottlenecks in clinical application that need to be broken through urgently. Clinical trials of some targeted drugs have not met expectations, and there is non-negligible drug toxicity—for example, although the FGFR inhibitor erdafitinib has brought new options for patients with FGFR mutations, the objective response rate is only about 40%, the long-term response durability is limited, and adverse events such as hyperphosphatemia often occur during treatment (30). The nectin-4 antibody–drug conjugate enfortumab vedotin has toxicity issues such as peripheral neuropathy, and some patients even develop primary drug resistance (31). In addition, the standardization challenge of biomarker testing seriously restricts the precision of targeted therapy. Taking FGFR mutation testing as an example, the consistency of the results from different testing platforms is poor, resulting in some potential benefit patients being missed and some ineligible patients receiving ineffective treatment.

The future development of BC-targeted therapy presents a trend of combination and diversification—for example, in a recent phase III clinical trial, 484 patients with locally advanced or metastatic urothelial cancer were randomly divided into the disitamab vedotin plus toripalimab group or the chemotherapy group. The results showed that the progression-free survival (PFS) of the disitamab vedotin plus toripalimab group was 13.1 months, the overall survival (OS) was 31.5 months, and the objective response rate (ORR) was 76.1%, all significantly better than those of the chemotherapy group, with fewer grade 3 or higher treatment-related adverse events (32). Another phase III clinical trial showed that after 344 participants with muscle-invasive bladder cancer were randomly assigned to receive enfortumab vedotin plus pembrolizumab or surgery alone, the event-free survival of the enfortumab vedotin plus pembrolizumab group was 74.4%, the OS was 79.7%, and the pathological complete response rate was 57.1%, which were significantly better than those of the surgery alone group (33). In addition, a literature review summarized the efficacy of poly(adenosine diphosphate [ADP]) ribose polymerase (PARP) inhibitors combined with programmed death ligand 1 (PD-L1) targeting in recent clinical trials, which represents a compelling strategy for treating BC with alterations in homologous DNA repair genes (34). Studies have shown that the mTOR pathway plays a role in both tumor cell growth and metabolism and T cell differentiation and activation. Rapamycin, as an mTORC1 inhibitor, has a dual effect: high doses directly inhibit tumor growth, while low doses enhance T cell immunity, providing a theoretical basis for the application of mTOR inhibitors combined with immunotherapy in BC patients (35). These studies all embody the concept of combination therapy for targeted therapy. The significant anti-tumor activity under combination therapy has brought new treatment opportunities for patients with advanced BC. The future development of BC-targeted therapy also presents a trend of precision. Precision treatment technologies based on synthetic biology provide new approaches to overcome existing challenges—for example, a research team modified the nuclease TnpB encoded by the IS200/IS605 transposon and its ωRNA (TnpB-ωRNA) scaffold to create an enhanced TnpB system (enTnpB system) and developed an AAV-ImmunAct immune activation protocol based on the enTnpB system, overcoming the obstacle that the CRISPR–Cas system is difficult to deliver due to the large size of the Cas protein (36). Another review of research on gene circuits in intelligent biotherapy for BC found that gene circuits, as a new type of precision tumor treatment, have advantages such as modularity, strong druggability, and short research and development cycles. They can recognize and integrate multiple molecular signals of tumors, induce the specific death of tumor cells through effector modules, reshape the immune microenvironment, and achieve efficient and controllable anti-tumor effects (37). Previous studies have shown that intratumor heterogeneity (ITH) affects the anti-tumor immune response and the efficacy of immunotherapy, and cellular MYC (c-MYC) is a key factor regulating the progression of ITH. The research team developed a c-MYC-based sensing circuit and a cell-to-cell communication system, which can break through the limitation of ITH to precisely target tumor cells and trigger a strong immunotherapeutic response (38). Through strategies such as precision optimization, more specific killing of BC tumor cells can be achieved.

However, several limitations must be acknowledged. Firstly, to ensure data operability, the study’s search timeframe was capped at May 2024. This temporal limitation may affect the completeness of our results, as important studies published after this cutoff date were not included. Secondly, the heterogeneity in data export formats across different databases posed challenges in ensuring comparability of results. To address this issue, we utilized only a subset of CiteSpace functionalities, which limited the depth of our analysis. Additionally, the incompatibility of data formats restricted our ability to employ other bibliometric tools such as VOSviewer for more comprehensive analyses. Lastly, due to the language capabilities of our team, this study primarily focused on English and Chinese literature, omitting publications in other languages, which introduced a potential bias by excluding potentially valuable contributions from non-English and non-Chinese sources. Future studies should strive to include a broader range of articles to ensure a more inclusive and comprehensive evaluation of the global research effort in BC-targeted therapy.

In conclusion, this pioneering bibliometric analysis has provided critical insights into the research landscape of targeted therapies for BC. The insights generated from our study will serve as a critical reference for researchers and clinicians aiming to investigate the mechanism underlying BC progression and enhance survival outcomes through precision medicine.

## Supplementary material

Supplementary Figure 1 Keywords analysis within the field. **(A)** Cluster analysis of keywords in English publications. **(B)** Cluster analysis of keywords in Chinese publications. **(C)** Timeline chart of keywords in English publications. **(D)** Timeline chart of keywords in Chinese publications. **(E)** Top 10 burst keywords in English publications. **(F)** Top 10 burst keywords in Chinese publications. Different colors represent different clusters in **(A)** and **(B)**. Blue lines indicate time intervals, and red lines indicate active periods in **(E)** and **(F)**.

Supplementary Table 1 Top 10 most productive journals in English and Chinese publications within this field.

Supplementary Table 2 Top 10 most cited articles in English and Chinese publications within this field.
