# Supplementary figures and images for: The international and domestic research trends of targeted therapy in bladder cancer: a bibliometric analysis of the past three decades
Source: Front Oncol. 2026 May 25;16:1850175. doi: 10.3389/fonc.2026.1850175 (PMC13243127; doi:10.3389/fonc.2026.1850175)

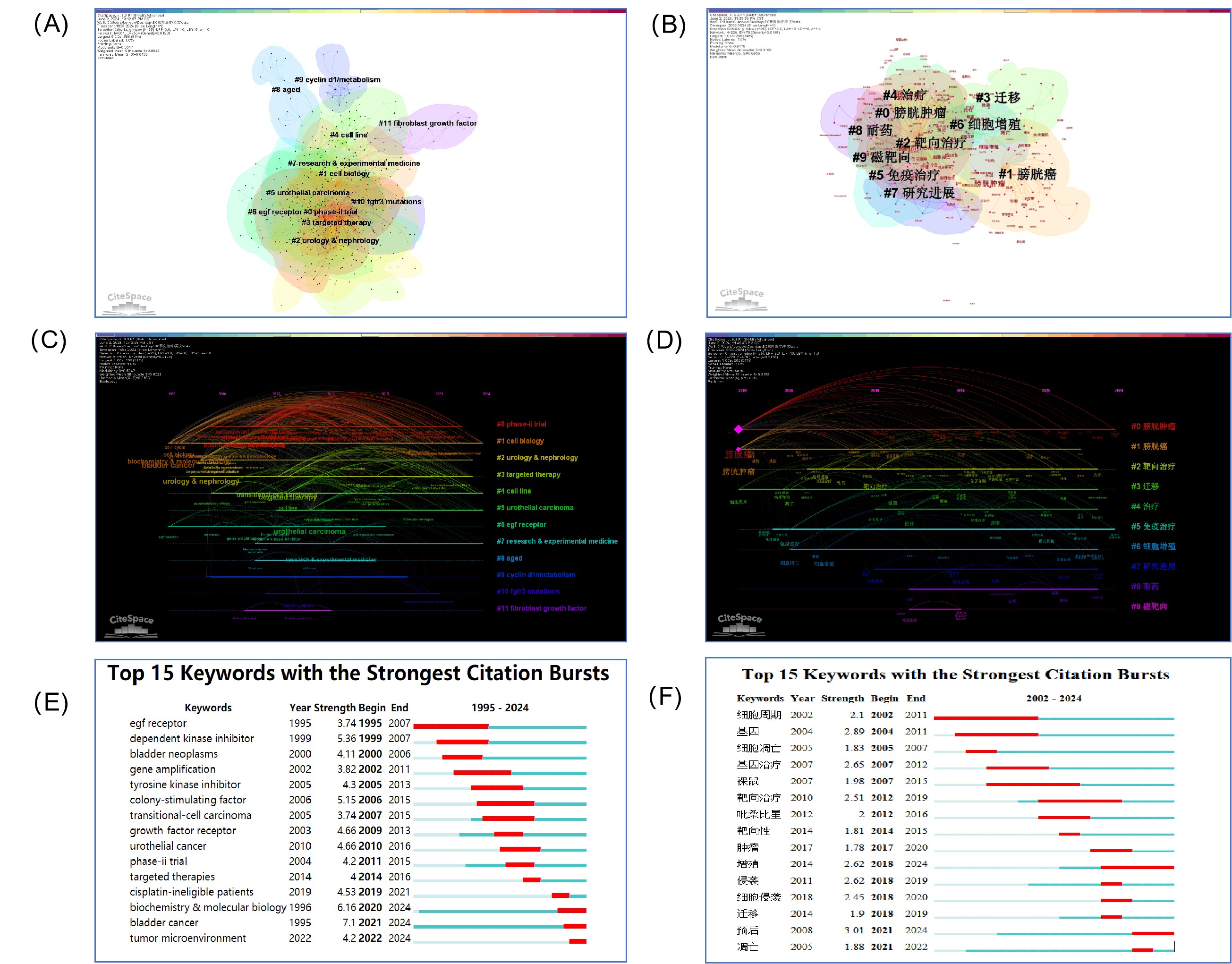

Supplement: Supplementary Figure 1 — Keywords analysis within the field. (A) Cluster analysis of keywords in English publications. (B) Cluster analysis of keywords in Chinese publications. (C) Timeline chart of keywords in English publications. (D) Timeline chart of keywords in Chinese publications. (E) Top 10 burst keywords in English publications. (F) Top 10 burst keywords in Chinese publications. Different colors represent different clusters in (A) and (B). Blue lines indicate time intervals, and red lines indicate active periods in (E) and (F). [file Image1.jpeg]
